# Nuclear morphology is a deep learning biomarker of cellular senescence

**DOI:** 10.1038/s43587-022-00263-3

**Published:** 2022-08-15

**Authors:** Indra Heckenbach, Garik V. Mkrtchyan, Michael Ben Ezra, Daniela Bakula, Jakob Sture Madsen, Malte Hasle Nielsen, Denise Oró, Brenna Osborne, Anthony J Covarrubias, M. Laura Idda, Myriam Gorospe, Laust Mortensen, Eric Verdin, Rudi Westendorp, Morten Scheibye-Knudsen

**Affiliations:** 1 Center for Healthy Aging, Department of Cellular and Molecular Medicine, University of Copenhagen, Copenhagen, Denmark; 2 Buck Institute for Research on Aging, Novato, CA USA; 3 Tracked.bio, Copenhagen, Denmark; 4 Methods and Analysis, Statistics Denmark, Copenhagen, Denmark; 5 Gubra, Hørsholm, Denmark; 6 Department of Microbiology, Immunology, and Molecular Genetics, David Geffen School of Medicine at UCLA, Los Angeles, CA USA; 7 Molecular Biology Institute, University of California, Los Angeles, Los Angeles, CA USA; 8 Laboratory of Genetics and Genomics, National Institute on Aging Intramural Research Program, National Institutes of Health, Baltimore, MD USA; 9 Istituto di Ricerca Genetica e Biomedica, Consiglio Nazionale delle Ricerche, Sassari, Italy; 10 Department of Public Health, University of Copenhagen, Copenhagen, Denmark

**Keywords:** Machine learning, Senescence, Ageing

## Abstract

Cellular senescence is an important factor in aging and many age-related diseases, but understanding its role in health is challenging due to the lack of exclusive or universal markers. Using neural networks, we predict senescence from the nuclear morphology of human fibroblasts with up to 95% accuracy, and investigate murine astrocytes, murine neurons, and fibroblasts with premature aging in culture. After generalizing our approach, the predictor recognizes higher rates of senescence in p21-positive and ethynyl-2’-deoxyuridine (EdU)-negative nuclei in tissues and shows an increasing rate of senescent cells with age in H&E-stained murine liver tissue and human dermal biopsies. Evaluating medical records reveals that higher rates of senescent cells correspond to decreased rates of malignant neoplasms and increased rates of osteoporosis, osteoarthritis, hypertension and cerebral infarction. In sum, we show that morphological alterations of the nucleus can serve as a deep learning predictor of senescence that is applicable across tissues and species and is associated with health outcomes in humans.

## Abstract (web summary)

Senescent cells are typically identified by a combination of senescence-associated markers, and the phenotype is heterogeneous. Here, using deep neural networks, Heckenbach et al. show that nuclear morphology can be used to predict cellular senescence in images of tissues and cell cultures.

## Main

Cellular senescence is widely recognized as a fundamental process in aging, both as a primary causal factor in the decline of tissue homeostasis and as a consequence of other aging processes such as inflammation and DNA damage^1–3^. Due to its critical role in disease etiology, senescence is increasingly recognized as a target for pharmaceutical intervention^4^. Senescence also serves as a biomarker for aging^5^, possibly providing a more nuanced measure of age-related health beyond chronological age. However, the role of senescence in human health is not clearly understood. Senescent cells present a complex and diverse phenotype, which varies substantially by cell type and source^6,7^. There is considerable overlap between molecular factors that associate with senescence, DNA damage, inflammation and other processes^8,9^, and no single marker reliably and consistently identifies senescence^10–12^. Importantly, senescent cells often exhibit an altered morphology, including expanded nuclei^13,14^, making senescence amenable to analysis with computer vision and machine learning methods^15^.

We present deep learning models that can predict cellular senescence with high accuracy based on nuclear morphology. Notably, predicted senescence correlates substantially with senescence-associated β-galactosidase (SA-β-gal), p16^Ink4a^, p21^Cip1^, p53 and DNA damage markers γH2AX and 53BP1 foci counts. Our senescence predictor was developed using normal human fibroblasts but also identifies increased senescence for multiple types of premature aging diseases, including Hutchinson–Gilford progeria syndrome, ataxia telangiectasia and Cockayne syndrome. We also evaluated mouse astrocytes and neurons and found increased senescence in cells subjected to ionizing radiation (IR), confirming its relevance to different cell types and organisms. These methods were further applied to H&E-stained mouse liver and human dermal tissues, predicting an increase in senescence with age. Using the Danish National Patient Register, which records all ambulatory and inpatient contacts with Danish hospitals, we investigated how predicted senescence relates to human disease. In our study of 169 individuals, we found a significant inverse relationship between malignant neoplasm incidence and predicted senescent cells, which fits the hypothesis that senescence is a mechanism to limit cancer^16–18^. Although oncogenic events are associated with the formation of senescent cells^17^, we speculate that individuals with higher propensity toward developing senescent cells have reduced formation of malignant neoplasm and are at lower risk of cancer. We also found weaker associations between predicted senescence and other conditions, including osteoporosis, osteoarthritis, hypertension, cerebral infarction, hyperlipidemia, hypercholesteremia, hearing loss, dyspnea and sciatica.

## Results

**Fig. 1:**
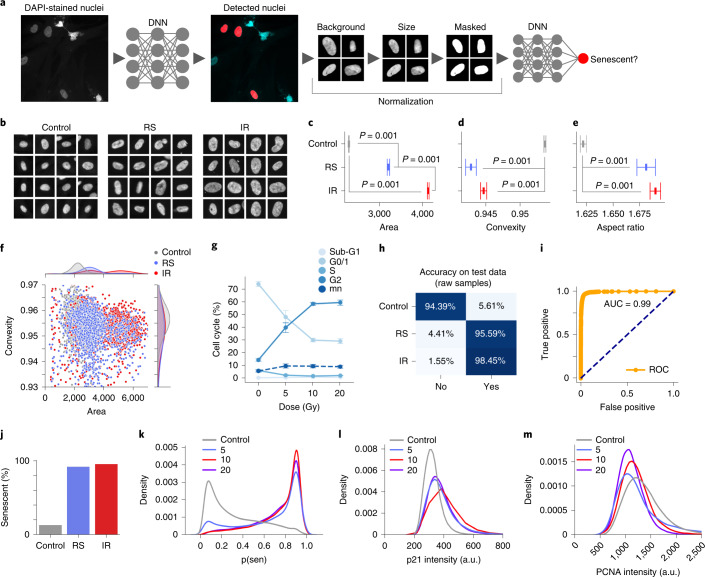
Nuclear morphology is an accurate senescence predictor in cultured cells. **a**, Analysis workflow. DNN, deep neural network. **b**, Sample nuclei for control, RS- and IR-induced senescent cells. **c**, Area of identified nuclei (RS *n* = 6,976, IR *n* = 19,193, control *n* = 68,971; mean ± 95% confidence interval (CI), Tukey multiple comparison). **d**, Convexity of identified nuclei (same as panel **c**). **e**, Aspect ratio of identified nuclei (same as panel **c**). **f**, Scatter plot of individual nuclei, with overall distributions for each at the top and right margins. **g**, Cell cycle analysis after exposure to several doses of IR; mn, multinucleated cells (*n* = 4, mean ± 95% CI). **h**, Accuracy of a deep neural network predictor on test data. **i**, Receiver–operating characteristic (ROC) curve of the deep neural network. **j**, Percentage of nuclei in each state classified as senescent for independent cell lines. **k**, Distribution of prediction probabilities for several doses of IR for three fibroblast cell lines. p(sen), predicted senescence score. **l**, Distribution of p21 intensities for several doses of IR for three fibroblast cell lines. **m**, Distribution of PCNA intensities for several doses of IR for three fibroblast cell lines.

**Extended Data Fig. 1:**
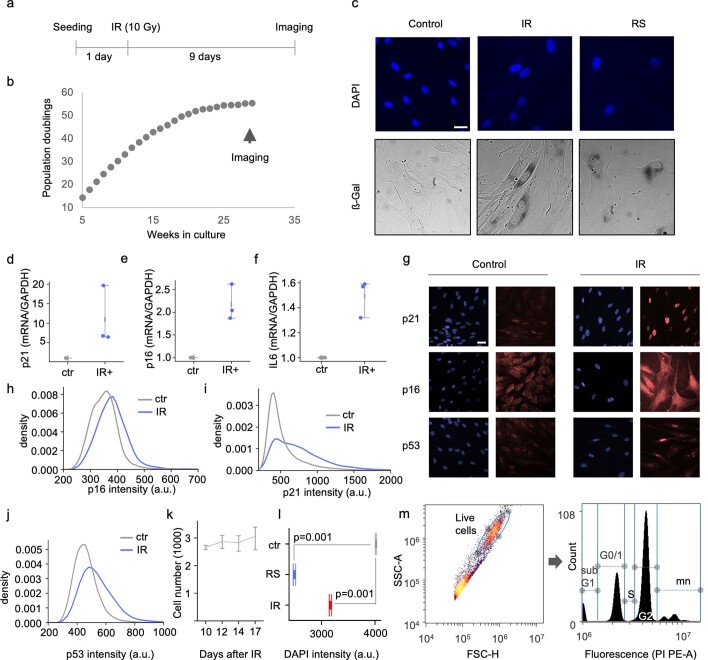
Cell culture models of senescence. **a** Schematic of Ionizing radiation (IR)-induced senescence. **b** Representative growth curve of cells undergoing replicative senescence (RS). **c** Representative micrographs of SA-β-gal activity and DAPI in control, IR and RS cells; scale bars, 10 μm. **d** Relative p21 mRNA expression levels by qPCR (n = 3, ± SEM). **e** Relative p16 mRNA expression by qPCR (n = 3, ± SEM). **f** Relative IL-6 mRNA expression by qPCR (n = 3, ± SEM). **g** Representative immunohistochemistry micrographs of nuclei with p21, p16, and p53 staining in control and IR cells; scale bars, 10 μm. **h** Distribution of p16 intensities for IR and control for three fibroblast cell lines (n = 9,196-27,716). **i** Distribution of p21 intensities for IR and control for three fibroblast cell lines (n = 13,678-32,730). **j** Distribution of p53 intensities for IR and control for three fibroblast cell lines (n = 12,844-31,100). **k** Cell count following irradiation (n = 3 cell lines, mean ± 95% CI). **l** DAPI intensities for IR, RS, and control for three fibroblast cell lines (RS n = 6,976, IR n = 19,193, control n = 68,971; mean ± 95% CI, two-sided Student’s t-test). **m** Gating strategy for cell cycle analysis (data shown is for 10 Gy treated fibroblasts), mn: multinucleated cells

Three dermal fibroblast cell lines were induced to senescence by IR or passaged until they reached replicative senescence (RS) (Extended Data Fig. 1a–c). To confirm that the IR-treated cells were senescent, we evaluated levels of senescence markers p16^Ink4a^, p21^Cip1^, p53 and interleukin-6 (IL-6) by immunohistochemistry and/or qPCR and found that IR led to a significant increase in these markers (Extended Data Fig. 1d–j). Importantly, IR induced growth arrest as measured by cell counts for 1 week after IR treatment (Extended Data Fig. 1k). Furthermore, 4,6-diamidino-2-phenylindole (DAPI) intensity has been shown to decrease with senescence^19^, and this was indeed the case in RS- and IR-treated cells (Extended Data Fig. 1l). Using these senescent models, DAPI-stained nuclei from IR and RS cells were imaged with a high-content microscope. Nuclei were detected using a deep convolutional neural network based on U-Net, which produced output images containing the detected nuclear regions. After extracting nuclei images, we applied several methods to normalize features, such as removing the background, standardizing the size of the nuclei and even masking inner details of the nuclei (Fig. 1a,b).

### Senescent cells display altered nuclear morphology

The morphology of the detected nuclei was analyzed to compare senescent cells to controls. There was a significant difference in nuclear area as previously reported^13^. Notably, IR senescent cells were also significantly larger than RS cells (Fig. 1c). Aging and certain premature aging diseases have been associated with greater irregularities or folds in the nuclear envelope^20,21^. We therefore evaluated the regularity of the nuclear envelope using convexity, a ratio of convex hull perimeter to perimeter. Convexity indicated that control cells were more regular compared to both IR and RS (Fig. 1d), and RS had the highest irregularity. This finding suggests convexity is another measure of senescence, with lower values corresponding to increased senescence. In addition, we found that both IR and RS had higher aspect ratios compared to control (Fig. 1e). We evaluated area and convexity per nuclei, observing overlapping clusters for the three states with area of RS overlapping both control and IR and convexity of RS and IR overlapping (Fig. 1f). Interestingly, the distribution of area for the IR senescent cells was bimodal, with the lower mode matching RS and a higher mode at almost twice the area, perhaps suggesting IR induced aneuploidy or stalling at the G2 checkpoint of the cell cycle. To further explore this hypothesis, we induced senescence with multiple IR doses and used flow cytometry to study the cell cycle. Remarkably, we observed a dose-dependent increase in G2 and corresponding loss of G1 and S-phase cells 10 days after the IR treatment (Fig. 1g and Extended Data Fig. 1m), indicating that IR induction leads to G2-stalled senescent cells as previously suggested^22,23^. Simple nuclear morphological measures appear to be a viable method for assessing cellular senescence in culture.

### Deep learning predicts senescence based on DAPI staining

**Extended Data Fig. 2:**
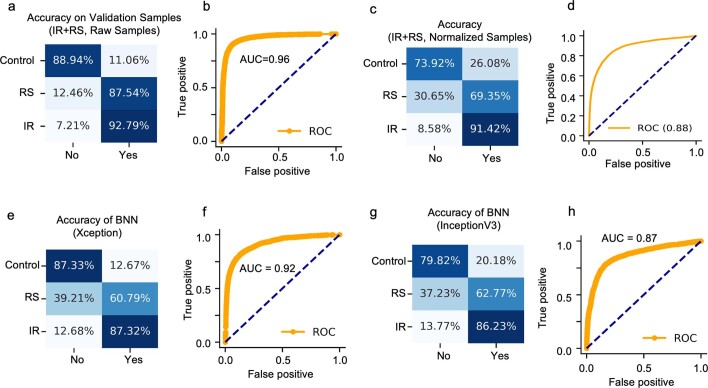
Performance characteristics. **a** Accuracy of raw model, tested on independent cell lines. **b** ROC curve of the raw model for independent cell lines. **c** Accuracy of normalized model. **d** ROC curve for the normalized model. **e** Accuracy of Xception-based Bayesian neural network. **f** ROC curve for the Xception BNN. **g** Accuracy of InceptionV3-based Bayesian neural network. **h** ROC curve for the InceptionV3 BNN.

Given the rich structure of nuclei, we applied deep neural networks to better assess senescence. Using Xception, one of the top performing models for image classification that has often been applied to biomedical classification^24,25^, we trained with 80% of the samples and held out 20% for testing, achieving accuracy of 95% (Fig. 1h,i; Supplementary Table 1 provides an overview of models throughout the paper). To eliminate any potential overfitting on the experimental context and cell lines, we evaluated an independent data set of two additional cell lines, giving accuracy of 94% (Extended Data Fig. 2a,b). The predictor indicates senescence for 12.7% of control, 92.0% of RS and 95.6% of IR (Fig. 1j).

To better characterize the performance with senescent phenotypes induced by multiple levels of stress, we applied the neural network predictor to cells exposed to different doses of radiation. All levels were predicted to be senescent, and there was a 9.7% mean increase between 5 and 10 Gy, but 10 Gy to 20 Gy show similar prediction scores (Fig. 1k). Proliferating cell nuclear antigen (PCNA) declines with increasing dose and p21^Cip1^ increases (Fig. 1l,m). Predicted senescence and the two markers align with experimental conditions, but the predictor appears to track p21^Cip1^ expression more closely. This experiment indicates that the treatment dose influences the senescent phenotype up to 10 Gy, a dose commonly used for senescence induction.

In sum, nuclear morphology represents a strong predictor of both replicative and DNA damage induced senescence.

### The deep predictor is confirmed by senescent markers

**Fig. 2:**
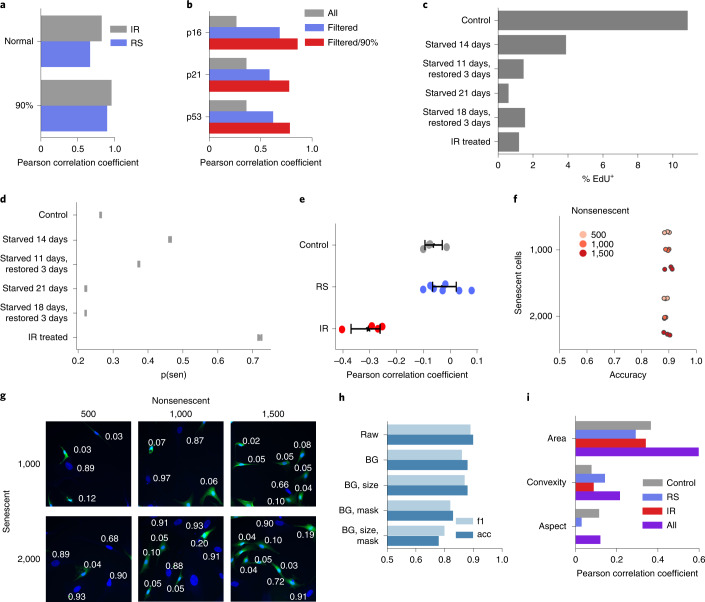
Analyzing quiescence, density, mixtures and morphology. **a**, Correlation between predicted senescence and nearby SA-β-gal regions, showing all and 90% confidence predictions only for RS and IR groups. **b**, Correlation between predicted senescence and multiple markers, showing all, filtered for markers with strong signals and filtered with 90% confidence predictions only. **c**, Percentage of EdU-positive cells for serum-starved quiescent, serum-starved and restored, IR-treated and control cells. **d**, Predicted senescence for quiescence, serum-starved, serum-starved and restored, IR-treated and control cells. **e**, Pearson correlation between density and predicted senescence (RS *n* = 7, IR *n* = 4, control *n* = 4; mean ± 95% CI). **f**, Prediction accuracy for several mixtures with different ratios of senescent and nonsenescent cells; each point represents mean accuracy for each of three cell lines. **g**, Samples of mixtures with different ratios of cells, showing predicted senescence per nuclei. **h**, Accuracy of deep neural networks trained and predicting after different normalization methods. acc, accuracy. **i**, Correlation between morphological metrics and predicted senescence by class. BG, background.

To confirm the accuracy of the predictor, we evaluated the correlation with several markers of senescence, including SA-β-gal, p16^Ink4a^, p21^Cip1^ and p53. Training a deep neural network to recognize SA-β-gal regions, we found SA-β-gal near nuclei for 64.1% of IR and 65.8% of RS compared to 19.6% for control, which roughly matches published rates for RS and controls^26^. A correlation analysis revealed a Pearson coefficient of 0.39 for IR and 0.31 for RS between predicted senescence and SA-β-gal detected nearby, but when restricting to the treated cells with nearby SA-β-gal and controls without it, the correlation rose to 0.83 for IR and 0.67 for RS (Fig. 2a). Applying a 90% confidence filter (see section below on deep ensemble methods), correlation rose to 0.96 for IR and 0.90 for RS, indicating the predictor is highly effective at recognizing senescence with detected SA-β-gal. Applying the same approach to classify p16^Ink4a^-, p21^Cip1^- and p53-positive cells led to correlation of 0.69 for p16^Ink4a^, 0.59 for p21^Cip1^ and 0.63 for p53. We also applied confidence filtering, restricting nuclei to those with high predictive confidence, and found correlation of 0.86 for p16^Ink4a^, 0.78 for p21^Cip1^ and 0.79 for p53 (Fig. 2b).

Cell cycle arrest is one of the primary characteristics of senescence, but this is also observed in quiescent cells. We therefore evaluated DNA synthesis with EdU staining in normal, serum-starved quiescent cells and IR-treated senescent cells. We found that in both quiescent and IR-treated groups, the percentage of EdU-positive cells was substantially lower than control (Fig. 2c). Importantly, IR-treated cells were predicted to be senescent, but serum-starved quiescent cells were not. However, shorter 14-day serum starvation showed some elevated senescence prediction, perhaps suggesting slight ambiguity of the predictor at this stage. Nevertheless, those remained below the 0.5 threshold, indicating that the predictor can discriminate between quiescence and senescence (Fig. 2d).

In cell culture, density can affect senescence, and confluent cells can evade senescence^27^. We evaluated how the predictor interprets nuclei with different densities, considering correlation between predicted senescence and density (number of nuclei per area). We found both control and RS groups had no correlation, but IR had a negative correlation (Fig. 2e), suggesting that more confluent cells may be resistant to IR-induced damage. We further evaluated the question of density and tissue culture heterogeneity by investigating if mixtures of senescent and nonsenescent cells in culture would affect the accuracy of the predictor. To do this, we stained proliferating cells with a cell tracer and co-cultured these cells with senescent cells. We examined different ratios across three cell lines and found that the predictor consistently distinguishes senescent cells from nonsenescent ones with mean accuracy of 89.2% and correlation between predicted senescence and the tracer of 0.87 (Fig. 2f,g). Importantly, there was no difference at all in senescence prediction regardless of the ratio of senescent versus nonsenescent cells. In total, the deep predictor is inferring senescence in agreement with multiple markers of senescence.

### Nuclear shape is a central predictive feature in senescence

It is unclear which aspects of nuclei images are used for assessment by the neural network. To investigate, we trained several models based on reduced forms of the images, such as masking the background, masking the nuclei itself, and scaling the nuclei to a standard size. With each reduction, we observed differences in classification accuracy on independent test lines (Fig. 2h). The background masking maintained accuracy of 88%, indicating limited reliance on the background. With background masked and size standardized, a trained model produced 88% accuracy, showing size differences played little role in senescence detection. This model was further reduced by completely masking the internal structure of the nuclei, which led to accuracy of 78% (Extended Data Fig. 2c,d). These experiments suggest that classification is largely based on the overall shape of the nuclei. We explored this further by evaluating Pearson correlation between predicted senescence and several morphological metrics, finding that area was moderately correlated (despite being standardized by the predictor) but convexity and aspect ratio were weaker (Fig. 2i). The deep learning model appears to be picking up on the nuclear shape in a more sophisticated manner than simple morphometrics.

The final reduced model yields an overall accuracy of 78%, and it shows an imbalanced per class accuracy of 73.9% for control, 69.3% for RS, and 91.4% for IR. Despite lower accuracy, the feature standardization and reduction make the model less influenced by technical variations such as image intensity, nuclear staining, magnification and others that could impact the utility of the predictor.

### Classification with confidence

Extending neural networks with Bayesian properties has several advantages, most notably providing a measure of confidence for predictions^28^. The Bayesian neural networks (BNNs) can be used to filter samples and reduce ambiguous predictions by requiring higher mean probability. Using FlipOut nodes^29^ in Tensorflow Probability, we converted Xception and InceptionV3 and trained using our senescent nuclei data set. Our BNN of Xception produced accuracy of 86% on raw images (Extended Data Fig. 2e,f), whereas the InceptionV3 BNN had accuracy of 80% (Extended Data Fig. 2g,h). The BNN models can thus be used to produce probability distributions of predicted senescence.

### A deep neural network ensemble increases predictive power

**Fig. 3:**
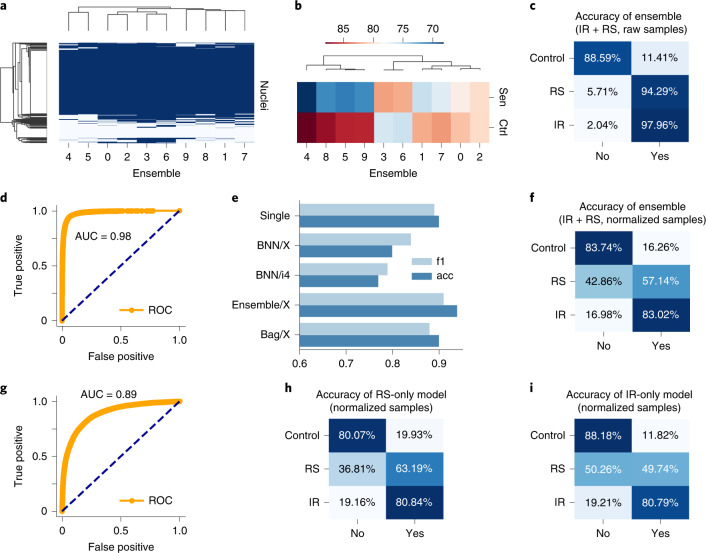
Predictions from deep ensembles increase accuracy. **a**, Heatmap of variation in predictions by members of ensemble; 500 sample nuclei as rows, ensemble members as columns; blue is control, and white is senescent. **b**, Heatmap of per-class accuracy for control (Ctrl) and senescent (Sen) by ensemble model. **c**, Accuracy of deep ensemble. **d**, ROC curve for the deep ensemble. **e**, Accuracy of single model, BNNs, deep ensemble and bagging (Bag). **f**, Accuracy of deep ensemble with normalized samples. **g**, ROC curve for the deep ensemble with normalized samples. **h**, Accuracy of RS-only model. **i**, Accuracy of IR-only model.

Exploring a large solution space during training, neural networks often select a relatively good solution that is biased^30^. Using an ensemble of deep models, the predictions can be combined to improve accuracy. To achieve this, we trained an ensemble with random initial weights, allowing convergence to different local minima. We found consistent agreement for the majority of samples; however, some show variation among the models (Fig. 3a). Evidently, some models balance the accuracy of each class in the middle of the range (75–80%), whereas other models skew toward one class at the expense of the other (for example, obtaining ~85% on one but ~70% on the other) (Fig. 3b). Although ensembles have benefits like a BNN, they can be less biased as each ensemble member can specialize around a solution, whereas a BNN is confined to local minima in solution space. Accordingly, we obtained good results with the ensemble method, finding accuracy of 94% (Fig. 3c,d). The BNN models can be used for confidence estimation but sacrifice performance, whereas the ensemble models give confidence estimation and improve performance (Fig. 3e). We therefore further evaluated the deep ensemble method with masked and normalized samples, giving 82% accuracy (Fig. 3f,g).

To help determine the type of senescence, we trained deep models on limited data, such as control versus RS or control versus IR to assess each type of senescence separately. Both models classified IR with high accuracy, but the RS model recognized RS with ~13% higher accuracy, whereas the IR model misclassified this portion of RS cells as control (Fig. 3h,i). Ensembles of deep neural networks provide greater accuracy for senescence prediction.

### Filtering by confidence increases accuracy

**Extended Data Fig. 3:**
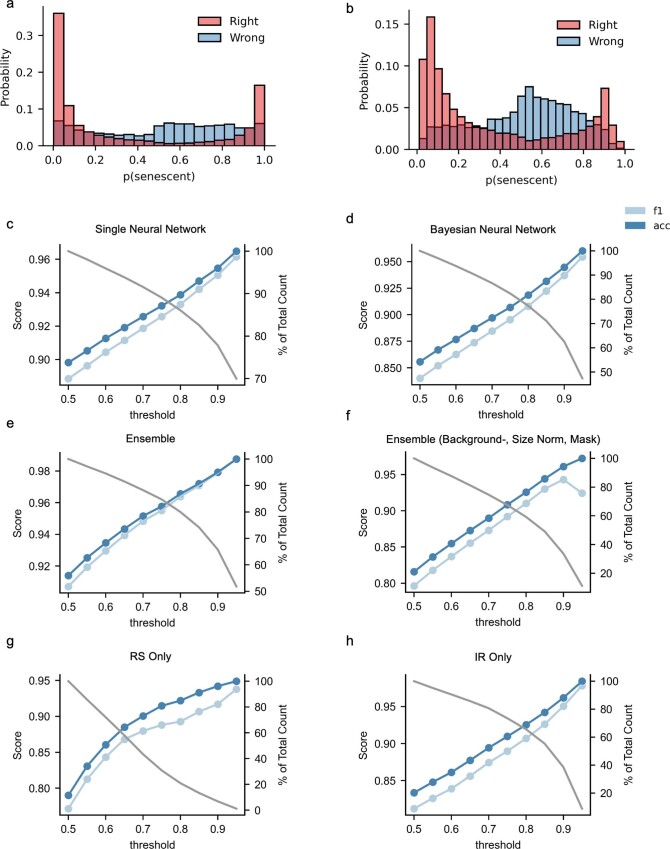
Improving performance by adjusting thresholds. **a** Histogram of predicted probabilities for Bayesian neural network. **b** Histogram of predicted probabilities for deep ensemble. **c** Accuracy and percent of samples evaluated with different classifier thresholds for single model. **d** Accuracy and percent of samples evaluated with different classifier thresholds for Xception-based Bayesian neural network. **e** Accuracy and percent of samples evaluated with different classifier thresholds for deep ensemble. **f** Accuracy and percent of samples evaluated with different classifier thresholds for deep ensemble with normalized samples. **g** Accuracy and percent of samples evaluated with different classifier thresholds for ensemble of RS-only for normalized samples. **h** Accuracy and percent of samples evaluated with different classifier thresholds for ensemble of IR-only for normalized samples.

To evaluate the accuracy of the models^28^, we sampled from the BNN or deep ensemble to determine their uncertainty predictions (Extended Data Fig. 3a,b). Correct predictions are oriented toward the lower and higher range of the output, representing greater certainty about samples’ states, whereas incorrect predictions tend towards the 0.5 threshold. We can therefore assume higher confidence in a model’s predictions by removing the predictions in the middle using thresholds. We evaluated a range of thresholds with several models (Extended Data Fig. 3c–f), which show a substantial increase in accuracy due to the ambiguous samples being discarded, including the ensemble of normalized models reaching accuracy of 97.2%. A similar approach was applied to other models, including the IR and RS models (Extended Data Fig. 3g,h), raising accuracy by 10–15%, although this reduces the number of cells considered.

### Predictor tracks development of the senescent phenotype

**Extended Data Fig. 4:**
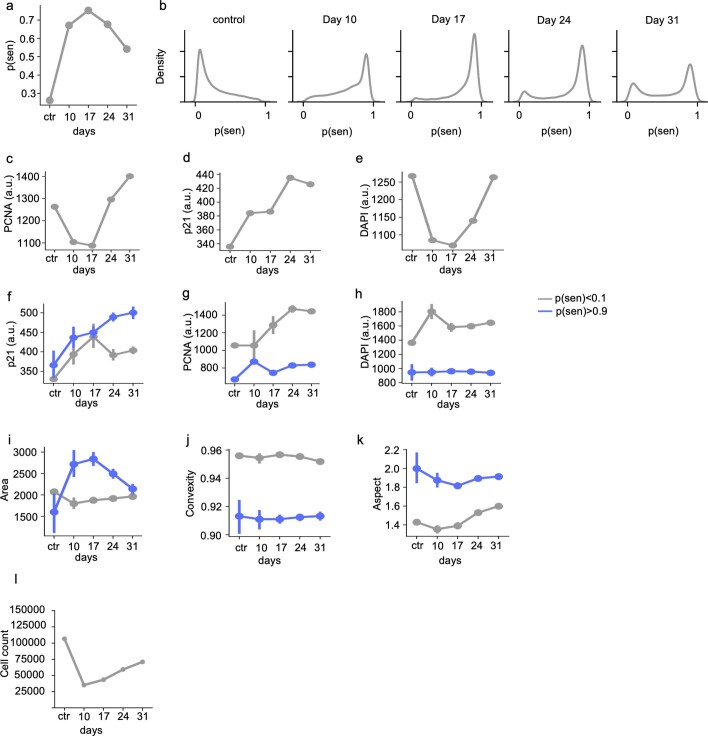
Development of Senescent Phenotype. **a** Predicted probability of senescence for several time points of IR-induced senescent cells for three fibroblast cell lines (n = 35,191-106,549). **b** Distribution of predicted probability split by time points. **c** Mean PCNA intensity per nucleus for several time points for three fibroblast cell lines (n = 35,191-106,549). **d** Mean p21 intensity per nucleus for several time points for three fibroblast cell lines (n = 35,191-106,549). **e** Mean DAPI intensity per nucleus for several time points for three fibroblast cell lines (n = 35,191-106,549). **f** p21 for several time points, split by predicted senescence state (p(sen)<0.1 n = 15,011, p(sen)>0.9 n = 6626; mean ± 95% CI). **g** PCNA for several time points, split by predicted senescence state (same as **f**). **h** DAPI for several time points, split by predicted senescence state (same as **f**). **i** Area for several time points, split by predicted senescence state (same as **f**). **j** Convexity for several time points, split by predicted senescence state (same as **f**). **k** Aspect for several time points, split by predicted senescence state (same as **f**). **l** Cell counts for several time points.

To better understand the development of the senescent phenotype and how nuclear morphology changes over time, we analyzed human fibroblasts induced to senescence by 10 Gy IR and imaged at days 10, 17, 24 and 31. The predictor identifies senescence at all four times points with probability that increases from days 10 to 17 but declines by day 31 (Extended Data Fig. 4a). Interestingly, examining the probability distribution of the predictor it was apparent that a growing peak of nonsenescent cells appear after day 17, suggesting that a small number of cells were able to escape senescence induction and eventually overgrow the senescent cells (Extended Data Fig. 4b). Indeed, when investigating markers of proliferation, we see that over the time course, PCNA declines until day 17, after which the expression starts to return (Extended Data Fig. 4c). p21^Cip1^ follows an inverse pattern with stain intensity increasing initially and then declining slightly by day 31 (Extended Data Fig. 4d). We also saw a decrease in DAPI intensity for days 10 and 17, indicating senescence, but a reversion to control level by day 31 (Extended Data Fig. 4e). To confirm that the predictor accurately determined senescence even 31 days after IR, we evaluated if markers of proliferation and senescence correlated with predicted senescence. Accordingly, cells with predicted senescence had higher p21^Cip1^ levels, lower PCNA and lower DAPI intensities and vice versa (Extended Data Fig. 4f–h). Morphologically, area and aspect are higher for predicted senescence, whereas convexity is lower (Extended Data Fig. 4i–k). Finally, a simple nuclei count confirms growth, following IR treatment (Extended Data Fig. 4l). Overall, the senescence predictor captures the state during development in agreement with multiple markers and morphological signs.

### DNA damage foci and area correlate with predicted senescence

**Fig. 4:**
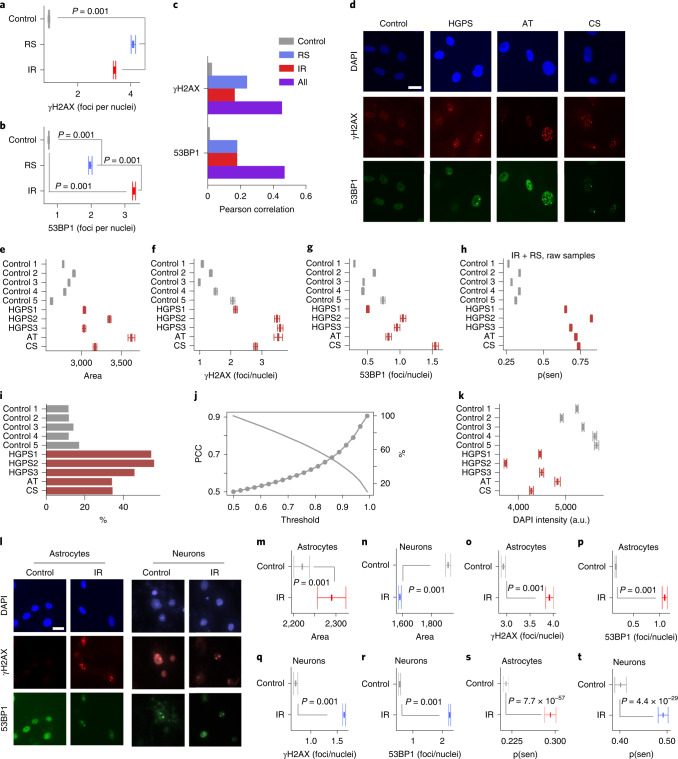
Senescence can be predicted across cell types and species. **a**, ɣH2AX foci per nuclei (RS *n* = 1,537, IR *n* = 5,365, control *n* = 9,971; mean ± 95% CI, Tukey multiple comparison). **b**, 53BP1 foci per nuclei (same as panel **a**). **c**, Correlation between foci count and predicted senescence. **d**, Representative micrographs; scale bar, 10 μm; HGPS, Hutchinson–Gilford progeria syndrome; AT, ataxia telangiectasia; CS, Cockayne syndrome. **e**, Nuclear area (AT *n* = 4,340, CS *n* = 4,524, P1 *n* = 9,948, P2 *n* = 4,924, P3 *n* = 4543, control 1 *n* = 14,480, control 2 *n* = 9,875, control 3 *n* = 15,074, control *n* = 9,371, control 5 *n* = 7,542, mean ± 95% CI). **f**, ɣH2AX foci per nuclei (same as panel **e**). **g**, 53BP1 foci per nuclei (same as panel **e**). **h**, Predicted probability of senescence per nuclei (same as panel **e**). **i**, Nuclei with nearby SA-β-gal regions. **j**, Correlation between predicted senescence and nearby SA-β-gal (left axis) and number of nuclei (right axis) with increasing thresholds. **k**, DAPI intensities (same as panel **e**). **l**, Representative micrographs of senescent murine astrocytes and neurons; scale bar, 10 μm. **m**, Nuclear area of murine astrocytes (IR *n* = 4,888, control *n* = 13,549; mean ± 95% CI, Tukey multiple comparison). **n**, Nuclear area of murine neurons (IR *n* = 62,847, control *n* = 33,303; mean ± 95% CI, Tukey multiple comparison). **o**, ɣH2AX foci per nuclei (IR *n* = 2,016, control *n* = 5,202; mean ± 95% CI, Tukey multiple comparison). **p**, 53BP1 foci per nuclei (same as panel **o**). **q**, ɣH2AX foci per nuclei (IR *n* = 63,241, control *n* = 33,533; mean ± 95% CI, Tukey multiple comparison). **r**, 53BP1 foci per nuclei (same as panel **q**). **s**, Predicted senescence (same as m). **t**, Predicted senescence (same as panel **q**). PCC, Pearson correlation coefficient.

**Extended Data Fig. 5:**
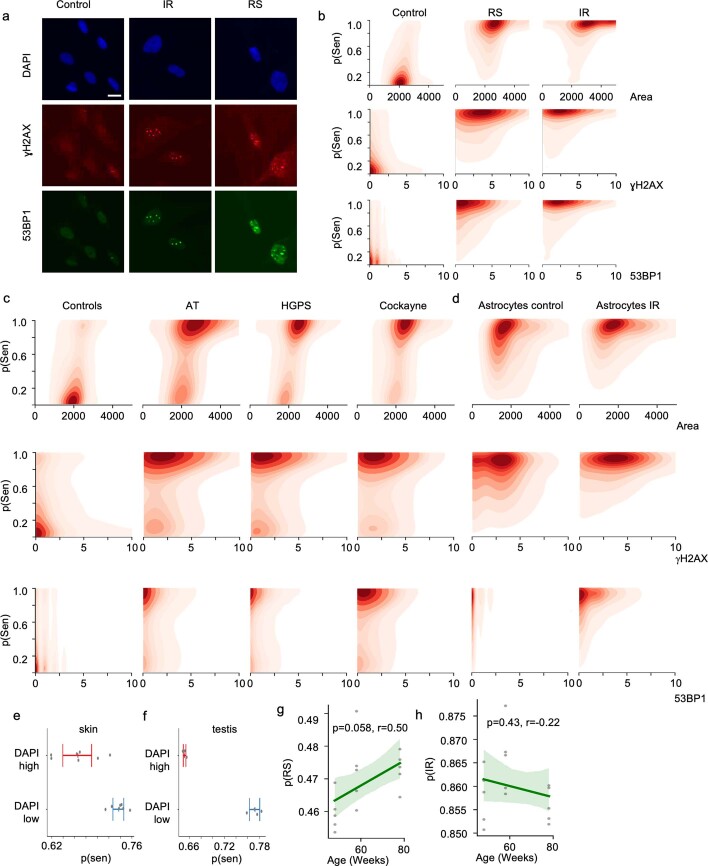
DNA Damage Foci and Other Senescence Indicators. **a** Representative immunohistochemistry micrographs of nuclei with DNA damage foci staining of gH2AX and 53BP1; scale bars, 10 μm. **b** 2D histogram (density heatmap) of predicted senescence and foci count per senescence type. **c** 2D histogram (density heatmap) of predicted senescence and foci count for premature aging diseases. **d** 2D histogram (density heatmap) of predicted senescence and foci count for murine astrocytes. **e** Predicted senescence of skin nuclei for high and low DAPI (DAPI low n = 7, DAPI high n = 7; mean ± 95% CI, two-sided Student’s t-test). **f** Predicted senescence of testis nuclei for high and low DAPI (DAPI low n = 4, DAPI high n = 4; mean ± 95% CI, two-sided Student’s t-test). **g** Predicted probability for RS senescence (n = 5, mean ± 95% CI, Wald Test with t-distribution). **h** Predicted probability for IR senescence (same as **g**).

Senescent cells are associated with the appearance of persistent nuclear foci of the DNA damage markers γH2AX and 53BP1 (refs. ^31,32^). Our base data set including control, RS and IR lines were examined for damage foci using high-content microscopy, where we found the mean count for controls to be below 1 for each marker, whereas RS had 4.0 γH2AX and 2.0 53BP1 foci and IR had 3.4 γH2AX and 3.0 53BP1 foci (Fig. 4a,b and Extended Data Fig. 5a). We calculated the Pearson correlation between predicted senescence and γH2AX and 53BP1 foci counts and found that across all conditions, there is a moderately strong correlation of around 0.5 (Fig. 4c). This association is also visible when simply plotting foci counts and senescence prediction, which shows predicted senescence flipping from low to high, along with shifts in foci counts (Extended Data Fig. 5b). Our feature reduction masked internal nuclear structure, but it is nonetheless notable that senescence prediction correlates with foci count. We also compared the correlation between predicted senescence and area, where we see a correlation of around 0.5. In sum, there is a considerable correlation between foci counts and senescence.

### Progeria cell lines display increased senescence

Patients with premature aging, or progeria, represent genetically well-defined models to understand the molecular basis of aging^33,34^. To test whether cell lines from patients with progeria display accelerated aging in culture, we applied the senescent classifier to primary fibroblasts isolated from Hutchinson–Gilford progeria syndrome, ataxia telangiectasia and Cockayne syndrome (Fig. 4d). Evaluating the area, we found that the nuclei of progeria cells are significantly larger than controls. Notably ataxia telangiectasia cells have the largest nuclei at 25% higher than controls, whereas Hutchinson–Gilford progeria and Cockayne syndrome are both 15% higher (Fig. 4e). We also investigated DNA damage foci and observe that most prematurely aged lines have higher γH2AX and 53BP1 foci counts (Fig. 4f,g and Extended Data Fig. 5c). Further, despite diverse mechanisms, the RS and IR ensemble classifier for raw images recognized these cell lines having greater probability of senescence (Fig. 4h). Evaluating SA-β-gal activity, we find 35–60% of nuclei have positivity and overall correlation of 0.5 between predicted senescence and having nearby SA-β-gal (Fig. 4i). When predictions are filtered to higher confidence levels, there is an increase in correlation up to 0.9 (Fig. 4j), indicating high-confidence predictions are capturing the senescent state. DAPI intensity also suggests that all progeria lines have higher senescence compared to controls (Fig. 4k). These observations indicate that our classifier may be able to discriminate rates of aging in cultured cells.

### Predictor translates across species and cell types in vitro

To broaden the applicability of our classifier, we speculated that it might apply to nuclei from other cell lines and species. We therefore evaluated the model on mouse primary astrocytes and neurons treated with IR (Fig. 4l). Although astrocytes are known to senesce with cell cycle arrest, post-mitotic neurons also exhibit a senescence-like state^35^. We first compared the nuclei area and found that the IR-treated astrocytes had slightly but significantly larger nuclei than controls, whereas IR-treated neurons had reduced area, unlike other cell types we studied (Fig. 4m,n). Evaluating DNA damage foci, we see that IR-treated astrocytes and neurons have substantially higher foci count as expected (Fig. 4o–r and Extended Data Fig. 5d). We next applied the ensemble of deep models (RS + IR model with full image normalization) and found that the IR-treated astrocytes had a 7.7% higher probability of senescence than controls (Fig. 4s). For neuronal cultures, it was necessary to include an additional step of image preprocessing to eliminate artifacts caused by dead cells sticking to the coating of the tissue culture plates. We restricted the size to a range that visually matched common neuron samples and found that IR-treated neurons were 9.0% higher than controls using the same model (Fig. 4t).

### Predictor translates across species and tissues in vivo

**Fig. 5:**
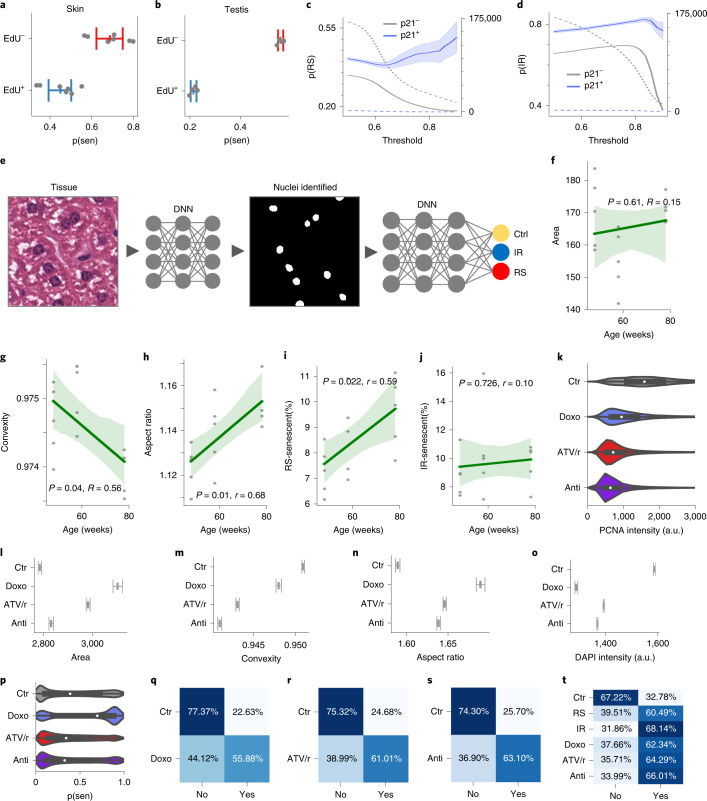
Senescence can be predicted across tissues and species. **a**, Predicted senescence for murine skin nuclei by EdU state (*n* = 7, mean ± 95% CI). p(sen), predicted senescence score. **b**, Predicted senescence for murine testis nuclei by EdU state (*n* = 4, mean ± 95% CI). p(sen), predicted senescence score. **c**, Mean probability of predicted RS senescence by p21 state across thresholds (mean ± 95% CI). p(RS), predicted RS senescence score. **d**, Mean probability of predicted IR senescence by p21 state across thresholds (mean ± 95% CI). p(IR), predicted IR senescence score. **e**, Analysis workflow. **f**, Nuclear area (*n* = 5 mice per group, mean ± 95% CI, Wald test with *t*-distribution). **g**, Nuclear convexity (same as panel **f**). **h**, Nuclear aspect ratio (same as panel **f**). **i**, Prediction percent for RS senescent (same as panel **f**). **j**, Prediction percent for IR senescence (same as panel **f**). **k**, PCNA intensity after senescence induction in three fibroblast cell lines (Doxo *n* = 30,957, ATV/r *n* = 119,669, Anti *n* = 106,920, Ctr *n* = 84,449; mean ± 95% CI). Doxo, doxocyline; ATV/r, atazanavir/ritonavir; Anti, antimycin A; Ctr, control. **l**, Nuclei area (as in panel **k**). **m**, Nuclei convexity (same as panel **k**). **n**, Nuclei aspect ratio (same as panel **k**). **o**, DAPI intensity (same as panel **k**). **p**, Predicted probability of RS senescence (same as panel **k**). **q**, Accuracy of doxorubicin-only model. **r**, Accuracy of ATV/r-only model. **s**, Accuracy of antimycin A-only model. **t**, Accuracy of unified model.

To investigate if the predictor could identify senescent nuclei in vivo, we injected mice with EdU to see if it would be able to distinguish proliferating cells from non-proliferating. We found that EdU positivity was strongly associated with low senescence scores, with a difference in mean scores of 23.7% for skin (*P* = 2.1 × 10^−4^) and 34.2% for testis (*P* = 2.6 × 10^−8^) (Fig. 5a,b). We also evaluated DAPI intensity, which showed lower levels for predicted senescence and a difference in mean prediction scores of 7.1% for skin and 12.6% for testis (Extended Data Fig. 5e,f). To determine the predictor’s ability to identify senescence in more slowly proliferating tissues, we stained liver tissue sections from mice with DAPI and p21^Cip1^, which can indicate senescence and increases with age in some tissues^36–38^. After identifying hepatocytes using image segmentation and fully normalizing nuclei images, we applied the model to predict senescence. We found that the mean predicted senescence per animal was significantly higher for p21^Cip1^-positive cells compared to p21^Cip1^-negative cells for both RS and IR models, which further increased as confidence filtering was applied (Fig. 5c,d).

We applied the predictor to H&E-stained liver tissue from C57Bl6 mice at taken at 48, 58 and 78 weeks of age. After imaging the tissue sections at ×20, we used a deep learning segmentation model trained on 18 tiles to extract nuclei from 16,187 tiles (Fig. 5e). Our training set included samples of hepatocytes only, and this cell type was primarily selected during automated segmentation. We first analyzed morphological metrics, finding an insignificant increase in nuclear area (Fig. 5f). However, we saw a significant decrease in convexity and increase in aspect ratio, both indicating increased senescence with age (Fig. 5g,h). Images of nuclei were normalized (scaled to the same size, background and internal details masked) and then evaluated for senescence using the RS and IR models. The RS model indicated increasing senescence with age, whereas the IR model did not (Extended Data Fig. 5g,h). Using the probability output, we calculated the percentage of senescent cells, finding ~36% for RS and ~99% for IR. The predictor is trained on DAPI-stained cultured fibroblasts representing a considerable difference in context, it is therefore likely that the algorithm should be tuned to evaluate other data sources. Applying thresholds of 0.6 and 0.9 for RS and IR, respectively, the percentage was brought down to around 8–10% to match the percentage reported, roughly adjusted for difference in age and split between IR and RS^39^. With these thresholds, the percentage of senescent cells per mouse increased with age (Fig. 5i,j). Given the differences in human and mouse nuclei as well as between cell types, it is notable that the senescent state can be captured through the relative difference in assessed probability. It therefore appears that our predictor may be able to determine senescence across cell types and species.

### Predictor recognizes senescence from multiple mechanisms

Senescence can be induced by several types of stressors that could result in different types of pathologies^40–42^. We therefore induced senescence by different drug treatments, including the DNA damaging compound doxorubicin, the mitochondrial toxin antimycin A and the HIV protease inhibitor atazanavir/ritonavir (ATV/r), and evaluated each of them. PCNA staining confirmed that each drug treatment led to a senescent state (Fig. 5k). A morphometric analysis showed that all three drug treatments expanded nuclear area, decreased convexity and increased aspect ratio (Fig. 5l–n). Additionally, DAPI intensity decreased significantly for all three treatments, indicating senescence (Fig. 5o). The predictor model (trained on IR and RS methods) recognized senescence in the nuclei treated with doxorubicin but did not detect senescence with antimycin A or ATV/r (Fig. 5p). We speculate that doxorubicin treatment more closely resembles the DNA damage caused by IR and RS. To address this limitation of our model, we trained new models for each new type, including doxorubicin-only, antimycin A-only and ATV/r-only images (Fig. 5q–s). In addition, we trained on a unified data set, including IR, RS, doxorubicin, antimycin A and ATV/r. Tested on validation data held out from training, we find the unified model can now recognize antimycin A with 66.0% accuracy, ATV/r with 64.3% accuracy and doxorubicin with 62.3% accuracy, which exceeds performance for each individual predictor (Fig. 5t). However, the model has reduced accuracy for IR at 68.1% (compared to 83.0%), although RS is slightly higher in this model. Although the base predictor model provides higher accuracy for IR, the unified model can recognize senescence in more diverse conditions.

### Predictor detects senescence in human dermis with p21^Cip1^

**Fig. 6:**
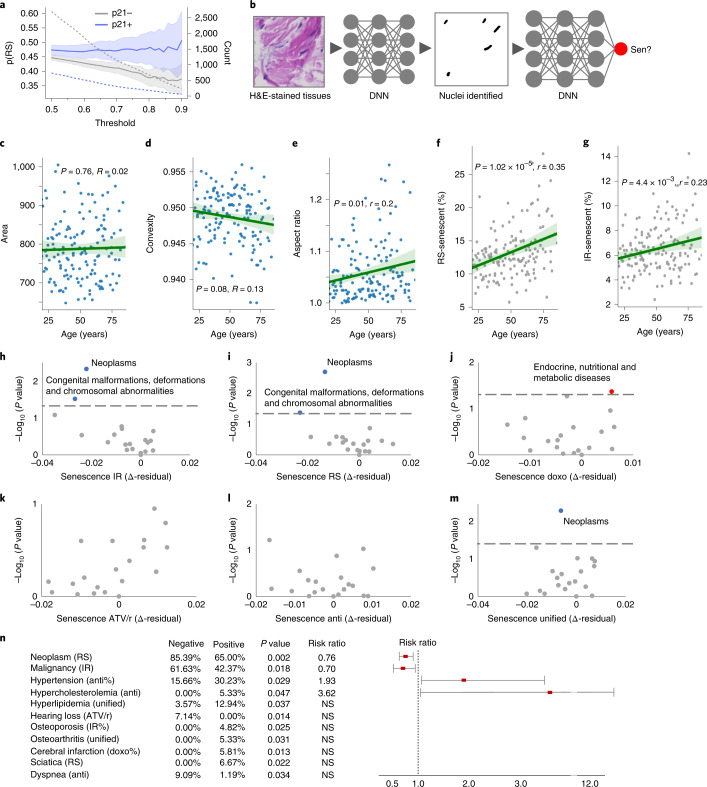
Nuclear morphology predicts senescence and multiple diseases in humans. **a**, Predicted probability of senescence by p21 state in human dermis, across thresholds (mean ± 95% CI). **b**, Analysis workflow. Sen, senescent. **c**, Nuclear area per patient (*n* = 148, mean ± 95% CI, Wald test with *t*-distribution). **d**, Nuclear convexity per patient (same as panel **c**). **e**, Nuclear aspect ratio per patient (same as panel **c**). **f**, Predicted percentage of RS senescence (*n* = 169, mean ± 95% CI, Wald test with *t*-distribution). **g**, Predicted percentage of IR senescence (same as panel **f**). **h**, Volcano plot of ICD-10 chapters based on IR senescence residuals and *P* values (two-sided chi-squared test). **i**, Volcano plot of ICD-10 chapters based on RS senescence residuals and *P* values (same as panel **i**). **j**, Volcano plot of ICD-10 chapters based on doxorubicin senescence residuals and *P* values (same as panel **i**). **k**, Volcano plot of ICD-10 chapters based on ATV/r senescence residuals and *P* values (same as panel **i**). **l**, Volcano plot of ICD-10 chapters based on antimycin A senescence residuals and *P* values (same as panel **i**). **m**, Volcano plot of ICD-10 chapters based on unified senescence residuals and *P* values (same as panel **i**). **n**, Disease conditions, percentage of individuals with negative or positive residuals, *P* value (two-sided chi-squared and Fisher’s exact tests) and risk ratio ± 95% CI. NS, not significant.

**Extended Data Fig. 6:**
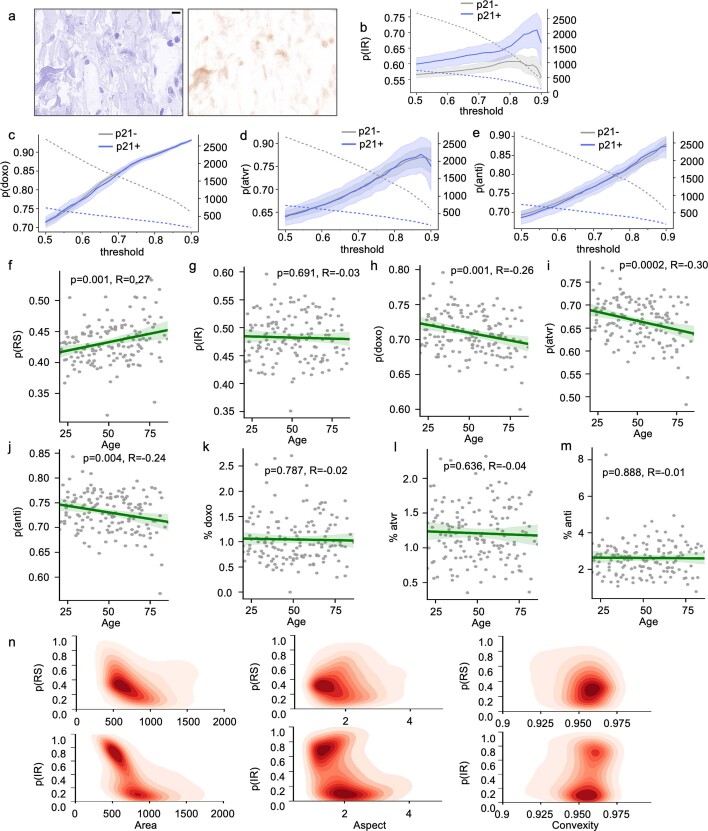
Senescence metrics in human dermal fibroblasts. **a** Sample of human dermis, stained with H&E and DAB for p21; scale bars, 10 μm. **b** Predicted probability of senescence from IR model and number of nuclei for p21- and p21+ nuclei in human dermis (mean ± 95% CI). **c** Predicted probability of senescence from Doxorubicin model and number of nuclei for p21- and p21+ nuclei in human dermis (mean ± 95% CI). **d** Predicted probability of senescence from ATV/r model and number of nuclei for p21- and p21+ nuclei in human dermis (mean ± 95% CI). **e** Predicted probability of senescence from antimycin-A model and number of nuclei for p21- and p21+ nuclei in human dermis (mean ± 95% CI). **f** Predicted probability of RS senescence (n = 169, mean ± 95% CI, Wald Test with t-distribution). **g** Predicted probability of IR senescence (same as **f**). **h** Predicted probability of doxorubicin senescence (same as **f**). **i** Predicted probability of ATV/r senescence (same as **f**). **j** Predicted probability of antimycin-A senescence (same as **f**). **k** Percent of doxorubicin senescence (same as **f**). **l** Percent of ATV/r senescence (same as **f**). **m** Percent of antimycin-A senescence (same as **f**). **n** 2D histograms (density heatmaps) of dermal nuclei.

To determine if the predictor could be used with human dermis, we analyzed samples from an independent data set^43^, stained with hematoxylin and p21^Cip1^ (Extended Data Fig. 6a). Nuclei were detected using image segmentation with U-Net trained on the hematoxylin nuclei. Images of nuclei were then extracted and fully normalized, and the predictor was used to generate senescence probability scores. After calibrating the p21^Cip1^ detection threshold to roughly match published rates, we found the mean predicted senescence of p21^Cip1^-positive nuclei was 5.9% higher than those without p21^Cip1^ for the RS and IR models (with *P* = 0.005 for both), whereas other models showed no difference (Fig. 6a and Extended Data Fig. 6b–e). As the confidence threshold was raised above the standard 0.5, p21^Cip1^ positivity was clearly separated from p21^Cip1^ negativity. With increasing confidence, the p21^Cip1^-positive nuclei generally showed higher predicted probability for IR, whereas the p21^Cip1^-negative nuclei showed lower predicted probability for RS. The percent difference between mean p21^Cip1^-positive and p21^Cip1^-negative probability also increased with higher confidence. Notably, all three other models (doxorubicin, ATV/r and antimycin A) showed no separation between p21^Cip1^ positivity and p21^Cip1^ negativity, indicating that they are picking up on other type-specific aspects of senescence.

### Human dermis shows an increase in senescent nuclei with age

**Extended Data Fig. 7:**
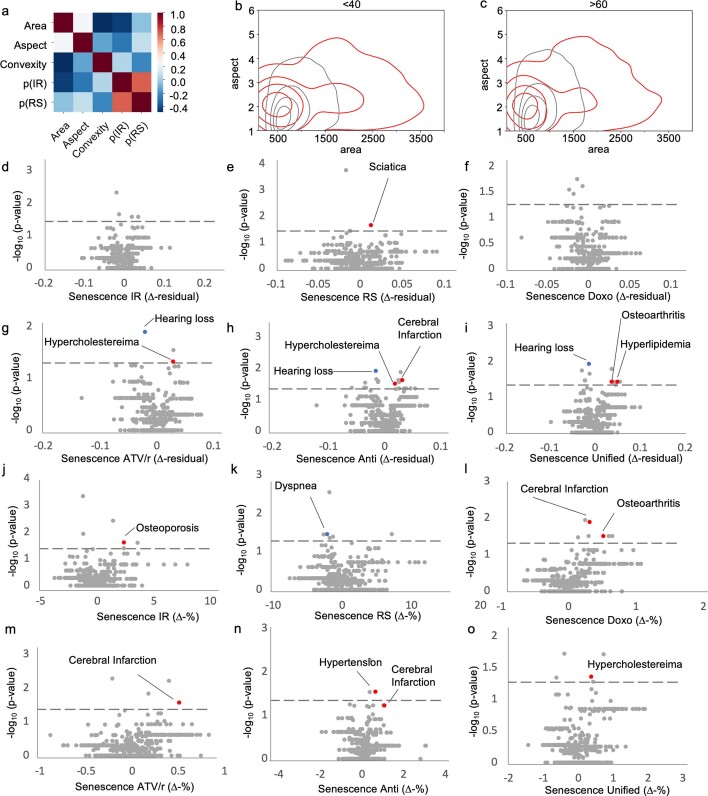
Additional Metrics and Clinical Codes. **a** Correlation coefficient matrix of metrics from nuclei. **b** Contour distribution of aspect versus area of nuclei for individuals under 40. **c** Contour distribution of aspect versus area of nuclei for individuals over 60. **d** Volcano plot of conditions based on IR senescence residuals and p values (two-sided Fisher’s exact test). **e** Volcano plot of conditions based on RS senescence probability residuals and p values (two-sided Fisher’s exact test). **f** Volcano plot of conditions based on doxorubicin senescence probability residuals and p values (two-sided Fisher’s exact test). **g** Volcano plot of conditions based on ATV/r senescence probability residuals and p values (two-sided Fisher’s exact test). **h** Volcano plot of conditions based on antimycin-A senescence probability residuals and p values (two-sided Fisher’s exact test). **i** Volcano plot of conditions based on probability of unified senescence probability residuals and p values (two-sided Fisher’s exact test). **j** Volcano plot of conditions based on percent IR senescence residuals and p values (two-sided Fisher’s exact test). **k** Volcano plot of conditions based on percent RS senescence residuals and p values (two-sided Fisher’s exact test). **l** Volcano plot of conditions based on percent doxorubicin senescence residuals and p values (two-sided Fisher’s exact test). **m** Volcano plot of conditions based on percent ATV/r senescence residuals and p values (two-sided Fisher’s exact test). **n** Volcano plot of conditions based on percent antimycin-A senescence residuals and p values (two-sided Fisher’s exact test). **o** Volcano plot of conditions based on percent unified senescence residuals and p values (two-sided Fisher’s exact test).

To further investigate if the predictor could be applied in a clinical context, we tested the algorithm on human skin samples of 169 individuals aged 20–86 years. The senescence classifier was used to evaluate the dermal nuclei from biopsy samples, stained with H&E and imaged in a slide scanner at ×20. We used U-Net to detect nuclei, extracted nuclear regions and fully normalized images of the nuclei (Fig. 6b). We first evaluated several morphological metrics, including area, convexity and aspect ratio. Across age, we see no change in area (Fig. 6c), an insignificant change in convexity (Fig. 6d) and a significant change in aspect ratio (Fig. 6e). We considered that different pathologies could be related to various forms of senescence (senescence caused by diverse mechanisms such as DNA damage, telomere attrition, mitochondrial dysfunction and so on). We therefore evaluated the multiple senescence predictor models developed and found the probability of senescence increases with age of patients for RS but is relatively flat for IR and declines for ATV/r, antimycin A and doxorubicin (Extended Data Fig. 6f–j). We expected a percentage of human dermal nuclei to be senescent, ranging from ~1% in young skin to ~15% in old skin^43^, so we selected thresholds to calibrate the model with 0.7 for RS and 0.85 for IR, leading to an overall predicted percentage of ~6% and showing an age-dependent increase in percent senescence (Fig. 6f,g). Both IR and RS models predict a statistically significant increase with age, whereas doxorubicin, antimycin A and ATV/r appear decoupled from age (Extended Data Fig. 6k–m). We also evaluated several morphological metrics and found RS was more correlated with convexity, whereas IR was more correlated with area and aspect ratio, perhaps indicating morphological aspects of each type of senescence in vivo (Extended Data Fig. 6n and 7a). Interestingly, we found that area was anti-correlated with both predicted IR and RS, but predicted IR was inverse to aspect ratio. This finding indicates differences between senescence in culture and in tissue sections and affirms that the IR and RS model are picking up on different aspects of senescence. We considered whether the age-dependent increase in predicted senescence could be related to change in proportions of detected cell types, so we compared the distribution of cell area and aspect ratio for broad age groups, individuals younger than 40 years and those older than 60 years. A shift in cell types should be reflected by a change in these metrics, but we found no noticeable difference in the distribution of these metrics for predicted senescent and nonsenescent cells between age groups (Extended Data Fig. 7b,c). Comparing each group by mean area and aspect ratio of individuals, a *t*-test showed nonsignificance (*P* = 0.94 and *P* = 0.51, respectively), indicating that each group has a similar proportion of cell types.

### Senescence is associated with multiple health conditions

**Extended Data Fig. 8:**
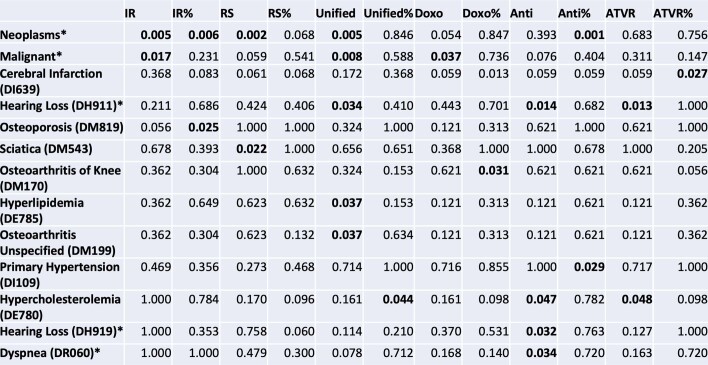
Table of Significant Conditions and Additional Conditions. Table of conditions with significant p values from two-sided chi-squared test (neoplasms and malignancy) or two-sided Fisher’s test (all others). Not corrected for multiple comparisons. * indicates negative association, others are positive.

Given the large variation in predicted senescence, we speculated that these values could represent meaningful health outcomes. To investigate this, we retrieved 19,820 International Classification of Diseases, 10th Revision (ICD-10) diagnosis codes collected in the Danish National Patient Register from 1977 to 2018 for all the individuals in the study. We looked for associations between individuals with diagnosed conditions grouped by ICD-10 chapters and predicted senescence above or below the age-dependent mean (those above or below the trendline using residuals from linear regression of predicted senescence versus age). We used the chi-squared test and Fisher’s exact test for the frequency of occurrence between the two groups (Fig. 6h–m). Remarkably, we found a significant correlation between a rate of senescence below the age-matched mean and the presence of ICD-10 Chapter II Neoplasm diagnosis codes for both RS and IR, with *P* values of 0.002 and 0.005, respectively. Narrowing down the analysis, we determined the association was based on malignant (versus benign or unknown) codes within ICD-10 Chapter II Neoplasm with IR *P* value at 0.018 and RS at 0.058. We also scanned individual ICD-10 clinical codes and found several other conditions associated with senescence, including osteoporosis, osteoarthritis, hypertension, cerebral infarction, hyperlipidemia, hypercholesteremia, sciatica, dyspnea and hearing loss, which were all significant when evaluated individually but nonsignificant when applying multiple test correction, such as the Bonferroni method (Fig. 6n and Extended Data Figs. 7d–o and 8a). All of these conditions were associated with higher levels of predicted dermal senescence, except for cancer, hearing loss and dyspnea, which were associated with lower levels. These conditions are drawn from different models; for example, neoplasms are particularly significant with RS, whereas hypertension only appears in the antimycin A model. Overall, we found that high assessed senescence corresponds to fewer neoplasms and malignancies while also indicating increased frequency of osteoporosis, osteoarthritis, hypertension and other conditions.

## Discussion

In this paper, we present a neural network classifier that can predict cellular senescence based on nuclear morphology. Trained on fibroblasts maintained in cell culture, the classifier achieves very accurate results, which was confirmed by applying it to independent cell lines. By training additional models on samples with reduced features, we infer that the shape of the nucleus alone provides a signal to indicate senescent state. DAPI-stained nuclei with background removed, size standardized and internal structure masked are still classified with high accuracy. These feature reduction methods serve a secondary purpose, making a model robust to technical variation; our neural network trained on reduced samples can make predictions on nuclei that were prepared in other experimental and imaging contexts. Indeed, the predictor distinguished senescent astrocytes and neurons, predicted an age-related increase in senescent liver cells and confirmed senescence in cell lines from patients suffering from premature aging. Although it is still debated if universal markers of senescence exist, our findings suggest that at least morphological alterations in nuclei may be common across some tissues and species.

We present several predictor models, including those that combine IR, RS and other methods, and those that specialize on each for improved accuracy. The base model trained on IR and RS can identify either type along with senescence induced by doxorubicin, indicating that the predictor has identified features found in multiple types related to DNA damage. Our base model did not accurately identify ATV/r and antimycin A, but a new model trained on all five methods could identify senescence induced by these diverse mechanisms. The unified model could be identifying a common signature or simply recognizing multiple phenotypes.

We evaluated the predictor with two tissues, mouse liver and human dermis, which include heterogenous cell types. It is possible that age-associated changes in cell types, such as an increase in tissue-resident macrophages, could influence the predictor. For the liver, this is likely not the case, as segmentation was specifically trained on the distinct nuclear morphology of hepatocytes. Nevertheless, age-associated changes in tissue composition should be considered when interpreting the outcome of the algorithm in vivo. In general, it is challenging to assess senescence in tissues owing to the lack of universal markers, the expression of senescence-associated markers by quiescent cells and some nonsenescent cell types and the diverse variations of senescence and the SASP. Although our deep learning models predict higher rates of senescence in p21-positive and EdU-negative nuclei and show an increased rate with age, their accuracy in tissues is unclear. Indeed, the true rate of senescence in tissues is poorly characterized and can perhaps only be approximated by combining multiple traditional markers. Accordingly, when evaluating tissues, we expect lower accuracy but the utility of the method can be increased by focusing on restricted cell types or evaluating cell-type proportions. Further, large image data, such as full slide images as well as a considerable number of samples, may be needed to address heterogeneity.

Given these caveats, our data show that individuals with a predicted higher rate of senescent cells have reduced neoplasms and malignant cancer. This finding is consistent with the notion that senescence is a mechanism to control cancer development by limiting uncontrolled proliferation^18^. Further, premalignant tumors express markers of senescence, which are absent in malignancies, and malignant tumors can regress and undergo senescence by switching off oncogenes^17^, supporting the protective role of senescence in blocking the progression of neoplasms to malignancies. In addition, loss of central senescence inducers such as p16^Ink4a^ is very common in many cancer types^44^. Of note, there is also evidence suggesting that cellular senescence promotes malignancy through the inflammatory senescence-associated secretory phenotype or SASP^45^, that senescent cells may appear in areas where tumors tend to subsequently develop^46^, and that senescent cells and SASP induced by cancer treatment led to worse survival and healthspan^47^. Although the role of senescence in cancer is highly complex, our results based on clinical data support the overall protective role for senescence in human health with regard to cancer. We also found several other conditions associated with senescence^48^, including osteoporosis^49^, osteoarthritis^50^, hypertension^51^, cerebral infarction^52^, hyperlipidemia, hypercholesteremia, dyspnea and sciatica.

We also investigated how our deep learning predictor results correspond to other measures of senescence. Nuclear area is known to expand during senescence^13,53,54^, and we confirmed this in our cell culture data set. On a per-nuclei basis, we found a moderate correlation between area and predicted senescence. However, due to our size standardization, it is unlikely this classic feature is the primary signal for our deep learning model. We also identified convexity and aspect ratio as indicators of senescence, finding moderate correlation with predicted senescence. Interestingly, we found no increase in area with age in the human dermis, but we found a significant increase in aspect ratio and a significant decrease in convexity, indicating that nuclei become stretched and irregular with advancing age in humans. These observations confirm that size standardization is necessary to generalize our neural network classifier and also demonstrate the value of our feature-neutral approach, where the neural network is trained to identify senescence from rich image data that is later reduced through feature removal.

In sum, our deep neural network model is capable of accurately predicting the senescent state from nuclear morphology using several imaging techniques and has been demonstrated with diverse applications. We applied the predictor to human skin samples and observed an age-dependent increase in senescence. Remarkably, individuals who appear to have higher rates of senescent cells show a reduced incidence of malignant neoplasms, supporting the hypothesis that senescence is a mechanism to limit cancer. Further, we find association between predicted senescent cell burden and other conditions, including osteoporosis, osteoarthritis, hypertension, cerebral infarction, hyperlipidemia, hypercholesteremia, dyspnea and sciatica. These findings support the idea of a dichotomy in aging, where senescence limits age-associated proliferation while promoting age-associated degeneration.

## Methods

All experiments and research conducted within this work comply with all relevant ethical regulations.

### Cell culture

All human-derived primary skin fibroblast cells were purchased from Coriell Institute. Control fibroblasts included AG08498 (male, 1 year), GM22159 (male, 1 day), GM22222 (male 1 day), GM03349 (male, 10 years) and GM05757 (male, 7 years). Cells were cultured at 37 °C and 5% CO_2_ either in 1:1 mix of DMEM GlutaMAX (Gibco, 31966047) and F-12 media (Gibco, 31765068) for AG08498, GM22159 and GM22222 or in EMEM media (Biowest, L0415-500) for GM03349 and GM05757. Fibroblasts derived from Hutchinson–Gilford progeria syndrome patients included AG06917 (male, 3 years), AG06297 (male, 8 years) and AG11513 (female, 8 years). Fibroblasts sampled from ataxia telangiectasia and Cockayne syndrome patients were GM03395 (male, 13 years) and GM01428 (female, 8 years), correspondingly. Cells were cultured at 37 °C and 5% CO_2_ in minimum essential medium (MEM) (Lonza, BE12-662F). Freshly isolated primary neurons and mouse astrocytes were kindly provided by the Department of Drug Design and Pharmacology, University of Copenhagen. Cells were cultured at 37 °C and 5% CO_2_ in DMEM GlutaMAX (Gibco, 31966047). All media were supplemented with 10% fetal bovine serum (FBS; Sigma-Aldrich, F9665) and 100 U ml^−1^ penicillin-streptomycin (Gibco, 15140163).

### Senescence induction

To achieve RS, control fibroblasts at early passages were seeded in T25 cell culture flasks (200,000 cells) and cultured over 32 weeks. After each splitting, cell number was recorded and population doubling level was calculated as log_2_(cell number during harvesting/cell number during seeding). The experiment was terminated when the population doubling level reached zero.

Induction of cellular senescence by IR, doxorubicin, antimycin A and ATV/r was performed according to Neri et al.^55^. Briefly, control fibroblast cells at early passages were seeded in 96-well plates (Corning, 3340) in a density of 2,000 cells per well. The day after, cells were either exposed to 10 Gy IR or treated with 250 nM doxorubicin for 24 h and cultured for the next 9 days. Medium was replaced every 2 days. Three days before radiated or doxorubicin-treated cells reached senescence state, fibroblast cells from the same stock were seeded (2,000 cells per well) as mock-irradiated or DMSO-treated controls. For tracking proliferating fibroblasts, control cells were stained with CFSE using Vybrant CFDA SE tracer kit (V12883, Thermo Fisher Scientific) according to the manufacturer’s protocol. The control cells were subsequently added to the senescent cells. The co-cultured proliferating and senescent cells were cultured for 24 h before fixing. Mitochondrial dysfunction-induced senescence was achieved by treating control fibroblast cells with 250 nM antimycin A every 2 days within 10 days. Then, 25 μM ATV/r was given to control fibroblast cells every 2 days within 14 days to develop senescence phenotype. Corresponding DMSO-treated controls were cultured in parallel and seeded in a 96-well plate 3 days before terminating the experiment. Temporary cell cycle arrest in quiescent fibroblasts was induced by culturing cells in 0.2% FBS-containing media for 14 and 21 days^56^. On day 11 for the 14-day treatment and day 18 for the 21-day treatment, media containing 10% serum was added back to half of cells to restore their proliferative capacity.

### Gene expression analysis

Radiated and control fibroblast cells were lysed using TRIzol reagent (Ambion by Life Technologies), and RNA was isolated using Direct-zol RNA miniprep Plus (Zymo Research). RNA was reverse transcribed into complementary DNA using High-capacity complementary DNA reverse transcription kit (Applied Biosciences) according to manufacturer’s protocols. Next, RT-qPCR was performed to detect mRNA levels of senescence markers using StepOnePlus Real-Time PCR System (Applied Biosystems). Relative gene expression was calculated based on obtained Ct values, normalized to housekeeping gene GAPDH and expressed as fold changes compared to nonirradiated control. The following specific primer sequences were used^55^: CDKN2A (p16^Ink4a^): forward: 5′-GAG CAG CAT GGA GCC TTC-3′, reverse: 5′-CGT AAC TAT TCG GTG CGT TG-3′; CDKN1A (p21^Cip1^): forward: 5′-TCA CTG TCT TGT ACC CTT GTG C-3′, reverse: GGC GTT TGG AGT GGT AGA AA; IL-6: forward: 5′-CAG GAG CCC AGC TAT GAA CT-3′, reverse: GAA GGC AGC AGG CAA CAC; GAPDH: forward: 5′-GTC AGC CGC ATC TTC TTT TG-3′, reverse: 5′-GCG CCC AAT ACG ACC AAA TC-3′.

In total, three independent sets were performed in GM22159 cells.

### Immunocytochemistry, SA-β-gal detection, EdU staining and image preparation

For detection of persistent DNA damage foci and fluorescence intensity levels of p16^Ink4a^, p21^Cip1^, p53 and PCNA, fibroblast cells were washed once with warm PBS and fixed in 4% paraformaldehyde solution for 15 min, followed by a permeabilization step with incubation for 10 min in PBS-0.1% Triton X-100. Blocking was performed in 1% BSA-PBS-0.1% Tween 20 overnight at 4 C. The next day, cells were incubated with primary antibodies (γH2AX, 1:1,000, Millipore, 05-636; 53BP1, 1:2,000, Novus, NB100-304; p16^INK4A^, 1:50, Santa Cruz Biotechnology, sc-56330; p21^Cip1^, 1:200, Santa Cruz Biotechnology, sc-6246; p53, 1:200, Santa Cruz Biotechnology, sc-126; PCNA, 1:500, Abcam, ab18197) for 1 h at room temperature, washed three times with PBS-T and incubated with secondary antibodies (1:200 Alexa Fluor 488, Invitrogen, 10424752; 1:200 Alexa Fluor 568, Invitrogen, 10348072) for 1 h at room temperature. Cells were incubated with DAPI solution (AppliChem, A4099) for 10 min and stored in PBS at 4 °C until the analysis.

EdU staining was detected using EdU Click 488 kit (Baseclick, BCK488-IV-IM-S) according to the manufacturer’s protocol. For all EdU-based experiments, cells were incubated with 10 μM EdU in cultured media for 1 h at 37 °C and 5% CO_2_. Cells were co-stained with DAPI solution (AppliChem, A4099) for 10 min. Plates were stored in PBS at 4 °C until the analysis.

SA-β-gal was detected using senescence cells histochemical staining kit (Sigma-Aldrich, CS0030) according to manufacturer’s protocol. Cell colonies were imaged using INcell analyzer 2200 high-content microscopy at ×20 magnification to produce 1199 images with 2,048 × 2,048-pixel resolution. Due to system constraints for object detection, each image was split into four tiles of 1,024 × 1,024-pixel resolution.

### Cell cycle analysis

Fibroblast cell lines AG08498, GM22159 and GM22222 were seeded in six-well cell culture plates, and 24 h later, DNA-damage induced senescence was conducted as described above (5, 10 or 20 Gy). After 9 days, cells were harvested by trypsinization and washed twice with PBS. Cells were fixed by adding dropwise ice-cold 70% ethanol while mixing the cells gently on a vortex mixer. Thereafter, the cells were incubated for 30 min on ice and washed twice in PBS. Fixed cells were incubated with RNase (100 µg ml^−1^; Thermo Fisher Scientific) at 37 °C for 30 min. Propidium iodide (20 µg ml^−1^; Sigma-Aldrich) was added and incubated for 30 min at 37 °C. Cell cycle status was determined by flow cytometry (CytoFlex, Beckman Coulter).

### Nuclei detection

A base library was prepared using control, irradiated (IR) and cells serially passaged until they reached senescence (RS). A deep neural network model was applied to detect DAPI-stained nuclei. The samples were used to build a training set for nuclei recognition. Several images were selected arbitrarily from each group for a total of ~20 samples, and all nuclei in the training samples were annotated by selecting the nuclear region. U-Net, a 23-layer fully convolutional network for image segmentation, was trained using the samples, learning to associate the DAPI images with annotation masks indicating nuclear regions. Our implementation of U-Net is largely based on the original U-Net^57^, but it includes a dropout layer after each of the convolutional and deconvolutional layers to reduce overfitting. After training for 1,000 epochs, the U-Net model was used to detect nuclei for all 4,796 tiles (1,199 images × four tiles/image), producing output images of predicted nuclei regions. The images with predicted nuclei were scanned for recognition regions of area between 500 and 15,000 pixels. Each detected nucleus was extracted along with its surrounding context as a centered 128 × 128-pixel region and used to assemble a base library of 95,152 nuclei. In addition, the recognition region itself was cutout, providing a two-color reduction of the detected nuclei, and assembled into a secondary library of nuclei masks.

### Nuclear morphology

An analysis of the nuclei was performed to assess morphological properties. The two-color mask library was used, as it provided a universal representation of the detected nuclei (with U-Net detector models that have good coverage of the nuclei region). Nuclear morphology was assessed using several metrics, including area, perimeter, moments, convexity, and aspect ratio. Convexity is the ratio of convex hull perimeter to perimeter, which provides a size-neutral measure of boundary regularity. The convex hull is a polygon that connects the outer edges of nuclei like an envelope.

### Senescent classification

After assembling a library of senescent cells, a deep neural network was trained to classify DAPI-stained nuclei as senescent or nonsenescent. The training set was based on several cell lines (GM22159, GM03349 and GM05757), whereas additional cell lines (GM22222 and AG08498) were used for testing. Training samples were randomized and split into 80% for training and 20% for validation. Due to experimental setup, the sample classes are unbalanced, with 75.2% control, 11.2% RS and 13.6% IR. The samples were balanced during training by applying class weights with inverse proportion to the class abundance (for example, senescent samples composed of IR and RS were fewer in number and therefore valued three times higher than controls). Image samples were normalized for brightness/intensity by adjusting each image’s mean intensity to 0 and standard deviation to 1. Augmentation was also applied during training, randomly modifying samples (adjusting size from 80% to 120%, changing normalized brightness from 70% to 130%, flipping horizontally and vertically and rotating up to 180 degrees). For each epoch, one augmentation cycle was performed. Training was done with Xception, a 48-layer model, initialized with ImageNet weights but set to allow weight adjustment of all layers during training. The top layer was replaced by a layer of one-hot nodes to indicate the state as controls or senescent. With this minor adjustment, the model provided 37,640,234 trainable parameters. Training was done using Adam with the learning rate set to 1 × 10^−4^ for ten epochs, in which time accuracy rapidly converged to a steady level. In addition, a simpler custom model was tested, with three convolutional layers with ReLU activation and two dense layers with L1/L2 regularization of 0.05/0.05 and 30% dropout. This model required 713,296 parameters. For both network designs, we trained with raw images along with several modified image sets, where the background was removed, the nuclei were size normalized and the inner details of nuclei were entirely masked (Fig. 1a). All three techniques were based on the detected nuclei. To remove the background, the area outside of the nuclei was set to 0. Size was normalized by rescaling all nuclei so the larger of the two dimensions was a standard size of 80 pixels. Finally, the size-normalized detection region was used for the masked nuclei set.

### BNN

We used Tensorflow Probability to create a BNN. We first converted the simple custom model, replacing nodes with the comparable FlipOut version^29^, which assumes that the kernel and bias are drawn from a normal distribution. During a forward pass, kernels and biases are sampled from posterior distribution. Targets were encoded as above, and the loss function used was cross-entropy plus Kullback–Leibler divergence divided by the number of batches. We also partially converted Xception to a BNN by replacing all dense and convolutional layers to FlipOut nodes, leaving separable convolutions unconverted, as a FlipOut version was not available. In addition, we fully converted InceptionV3 for evaluation. Inference was done by evaluating the model 20 times to produce a distribution of predictions and then taking the mean probability for each sample.

### Deep neural network ensemble

To improve accuracy and provide a more robust solution, we also worked with an ensemble of deep learning models. This method used ten models of Xception, each trained on the same data set with different random weight initialization. To generate predictions, each model instance was applied, and the results combined by taking the mean prediction. We also tried bagging, also known as bootstrap aggregation. Similar to the deep ensemble, this method trains different model instances with bootstrap selection of samples for *n* = 1 − 1/e. With each instance trained on a different subset of samples, this method produces multiple models that in theory can specialize to different sets of data.

### Software

The following software tools were used for the predictor and related analysis: python 3.8.10, tensorflow-gpu 2.4.1, tensorflow-probability 0.11.1, scipy 1.6.2, statsodels 0.12.0, scikit-learn 0.23.2, pandas 1.1.1, seaborn 0.11.1 and numpy 1.21.6, R 4.0.2

### Statistics and reproducibility

All comparisons between groups of samples were made using one-way analysis of variance *f*-tests to evaluate differences in the means, followed by pairwise tests using Tukey’s honest significant difference to calculate *P* values between groups. Linear regression methods were evaluated with R and *P* value statistics. Groups of patients were compared using the chi-squared test and Fisher test to detect significant differences between frequencies. Correlation was evaluated using the Pearson colocalization coefficient. No statistical method was used to predetermine sample size. No data were excluded from the analyses. The experiments were not randomized. The investigators were not blinded to allocation during experiments and outcome assessment. Data distribution was assumed to be normal, but this was not formally tested.

### Pathology sample selection

The individuals were sampled from patients for whom samples of naevi on non-sun-exposed skin had undergone pathology without malignant findings at a major pathology department in Copenhagen. The patient sample was selected to have flat distribution of age. We selected patient samples from the Danish National Register of Pathology requisitioned in 2007–2017 and coded with one or more PatoSNOMED topology code: T02530 (skin on penis), T76330 (foreskin), T80200 (mons pubis), T02471(skin on nates), T02480 (skin on abdomen), T02430 (skin on breasts) and one or more procedure code: P30620 (resect), P306X0 (ectomy preparation), P30611 (excision biopsy) and one or more morphology code: M87400 (junction naevus), M87500 (dermal naevus) and M87600 (compound naevus).

### Senescence and human morbidity

We collected ICD-10 diagnosis codes from the Danish National Patient Register in the period 1977-2018 of each of the patients in this study. We further grouped diagnoses into each of 21 ICD-10 chapters. We calculated the linear regression residuals of the relationship between age at pathology examination and the predicted senescent cell load (IR, RS metrics) for each of the patients. We then constructed contingency tables counting the number of patients with and without a specific diagnosis and with a predicted senescent cell load above or below the age-dependent average. We used Pearson’s chi-squared test and Fisher’s exact test to determine whether patients with a predicted senescent cell load above or below the age-dependent average were associated with a higher or lower incidence of specific diagnosis codes (or diagnosis within a specific ICD-10 chapter.)

### Animals

For liver histology studies, 15 male C57BL/6J mice were acquired from Janvier Labs. Animals arrived at 5–8 weeks of age and were housed in a controlled environment (12-h light/dark cycle, 21 ± 2 °C, humidity 50 ± 10%). Stratification and randomization into individual diet groups were based on baseline body weight. Mice had ad libitum access to tap water and chow (2018 Teklad Rodent Diet, Envigo; Altromin 1324, Brogaarden). Animals were aged to 48, 58 and 78 weeks, with five animals per group. For EdU proliferation in mouse tissues, six male and six female 12-month-old C57BL/6NRj mice (Janvier Labs) were bred in-house at the Department of Experimental Medicine, University of Copenhagen and maintained in a climate-controlled room with a 12-h light/dark cycle and ad libitum access to standard laboratory chow (Altromin 1324, Brogaarden) and water.

### Ethics

All animal experiments were approved by the Institutional Animal Care and Use Committee at MedImmune and/or the Danish Animal Experiments Inspectorate (licenses 2017-15-0201-01378 and 2017-15-0201-01321) and performed in accordance with internationally accepted principles for the use of laboratory animals including the European directive 2010/63/EU on the protection of animals used for scientific purposes. Work on human samples and clinical records were approved by the National Committee on Health Research Ethics (H-19078472) and the Danish Data Protection Agency (514‐0226/18‐3000).

### Liver histology

Terminal liver samples were dissected from the left lateral lobe immediately after killing the animal and subsequently fixed overnight in 4% paraformaldehyde. The liver tissue was then paraffin-embedded and sectioned at a thickness of 3 µm. Sections were stained with H&E (Dako), or DAPI (Invitrogen) co-stained with p21^Cip1^ (ab188224, Abcam). Slides were scanned by ScanScope AT System (Aperio).

### EdU cell proliferation assay

EdU incorporation in mice was assessed by intraperitoneal injection of 50 mg kg^−1^ EdU (Baseclick) dissolved in sterile PBS, and 4 h later, mice were euthanized and tissues collected. Testis and epididymis were dissected free from the epididymal fat pad, whereas dorsal skin was shaved, visible fat was removed and samples were fixed in neutral buffered formalin for 48 h. Sections were paraffin-embedded and sectioned by the Core Facility for Integrated Microscopy at the University of Copenhagen. Tissue sections were deparaffinized in xylene and rehydrated before fixation in 4% cold paraformaldehyde for 15 min, washed with PBS with 1% BSA and permeabilized in 0.5% Triton X-100 for 20 min. EdU staining was carried out according to manufacturer’s protocol (EdU In Vivo Imaging Kit 488, Baseclick), co-stained with 1 μg ml^−1^ DAPI and coverslips mounted with Prolong Gold Anti-Fade (Thermo Fisher Scientific). Whole slides were imaged on a Zeiss Axioscan.Z1 (Zeiss Microscopy), equipped with a Plan Apo 20×0.8 NA objective and a Semrock F66-887 multiband filter cube or a NanoZoomer-XR (Hamamatsu).

### Reporting summary

Further information on research design is available in the Nature Research Reporting Summary linked to this article.

## Supplementary information

Supplementary Information Supplementary Table 1

Reporting Summary

## Data Availability

Images of human dermis and medical record are obtained upon application from the Danish Pathology Register and cannot be shared by the authors due to medical privacy regulations. All other data are available from the authors upon reasonable request.

## Code availability

The code used in this study is available under https://github.com/scheibye-knudsen-lab/sen_predictor.
