# Supplementary material for: Nuclear morphology is a deep learning biomarker of cellular senescence
Source: Nat Aging. 2022 Aug 15;2(8):742–55. doi: 10.1038/s43587-022-00263-3 (PMC10154217; doi:10.1038/s43587-022-00263-3)
Supplement: Supplementary file 1 — Supplementary Table 1 [file 43587_2022_263_MOESM1_ESM.pdf]

nature portfolio

https://doi.org/10.1038/s43587-022-00263-3

Supplementary information

Nuclear morphology is a deep learning biomarker of cellular senescence

In the format provided by the authors and unedited

Figure	Model	Output Type	Data Standardization	Data Group	Accuracy	F1-Score	AUC
1H	X	Bi-state	Raw	Test 20%	95%	94%	0.99
Ext 2A	X	Bi-state	Raw	Ind 2 lines	94%	92%	0.96
N.A.	X	Bi-state	BgMask	Ind 2 lines	88%	86%	0.94
N.A.	X	Bi-state	BgMask+SizeStd	Ind 2 lines	88%	87%	0.94
Ext 2C	X	Bi-state	BgMask+SizeStd+Mask	Ind 2 lines	78%	80%	0.88
Ext 2E	X/BNN(partial)	Bi-state	Raw	Ind 2 lines	86%	84%	0.92
Ext 2G	InceptionV3/BNN	Bi-state	Raw	Ind 2 lines	80%	79%	0.87
3C	X/ensemble	Bi-state	Raw	Ind 2 lines	94%	91%	0.98
3F	X/ensemble	Bi-state	BgMask+SizeStd+Mask	Ind 2 lines	82%	80%	0.89

Supplementary table. An overview of the models used.
