# Supplementary material for: Nuclear morphology is a deep learning biomarker of cellular senescence
Source: Nat Aging. 2022 Aug 15;2(8):742–55. doi: 10.1038/s43587-022-00263-3 (PMC10154217; doi:10.1038/s43587-022-00263-3)
Supplement: Supplementary file 2 — Reporting Summary [file 43587_2022_263_MOESM2_ESM.pdf]

nature portfolio

Corresponding author(s): Morten Scheibye-Knudsen

Last updated by author(s): Jun 11, 2022

Reporting Summary

Nature Portfolio wishes to improve the reproducibility of the work that we publish. This form provides structure for consistency and transparency in reporting. For further information on Nature Portfolio policies, see our Editorial Policies and the Editorial Policy Checklist.

Statistics

For all statistical analyses, confirm that the following items are present in the figure legend, table legend, main text, or Methods section.

n/a	Confirmed	
☐	☒	The exact sample size (*n*) for each experimental group/condition, given as a discrete number and unit of measurement
☐	☒	A statement on whether measurements were taken from distinct samples or whether the same sample was measured repeatedly
☐	☒	The statistical test(s) used AND whether they are one- or two-sided<br>*Only common tests should be described solely by name; describe more complex techniques in the Methods section.*
☐	☒	A description of all covariates tested
☐	☒	A description of any assumptions or corrections, such as tests of normality and adjustment for multiple comparisons
☐	☒	A full description of the statistical parameters including central tendency (e.g. means) or other basic estimates (e.g. regression coefficient) AND variation (e.g. standard deviation) or associated estimates of uncertainty (e.g. confidence intervals)
☐	☒	For null hypothesis testing, the test statistic (e.g. F, t, r) with confidence intervals, effect sizes, degrees of freedom and P value noted<br>*Give P values as exact values whenever suitable.*
☒	☐	For Bayesian analysis, information on the choice of priors and Markov chain Monte Carlo settings
☒	☐	For hierarchical and complex designs, identification of the appropriate level for tests and full reporting of outcomes
☐	☒	Estimates of effect sizes (e.g. Cohen's d, Pearson's r), indicating how they were calculated

Our web collection on statistics for biologists contains articles on many of the points above.

Software and code

Policy information about availability of computer code

Data collection	No software was used for data collection
Data analysis	Cytobank; tensorflow-gpu 2.4.1, tensorflow-probability 0.11.1, scipy 1.6.2, python 3.8.10, statsodels 0.12.0, scikit-learn 0.23.2, pandas 1.1.1, seaborn 0.11.1, numpy 1.21.6, R 4.0.2

For manuscripts utilizing custom algorithms or software that are central to the research but not yet described in published literature, software must be made available to editors and reviewers. We strongly encourage code deposition in a community repository (e.g. GitHub). See the Nature Portfolio guidelines for submitting code & software for further information.

Data

Policy information about availability of data

All manuscripts must include a data availability statement. This statement should provide the following information, where applicable:

- Accession codes, unique identifiers, or web links for publicly available datasets
- A description of any restrictions on data availability
- For clinical datasets or third party data, please ensure that the statement adheres to our policy

Image data for most of this study is available upon reasonable request. Images of human dermis are restricted due to medical privacy regulation and are not available.

Field-specific reporting

Please select the one below that is the best fit for your research. If you are not sure, read the appropriate sections before making your selection.

☒ Life sciences ☐ Behavioural & social sciences ☐ Ecological, evolutionary & environmental sciences

For a reference copy of the document with all sections, see nature.com/documents/nr-reporting-summary-flat.pdf

Life sciences study design

All studies must disclose on these points even when the disclosure is negative.

Sample size	no sample size calculation has been performed
Data exclusions	no data was excluded
Replication	all experiments were replicated as indicated
Randomization	no randomization was performed
Blinding	no blinding was performed

Reporting for specific materials, systems and methods

We require information from authors about some types of materials, experimental systems and methods used in many studies. Here, indicate whether each material, system or method listed is relevant to your study. If you are not sure if a list item applies to your research, read the appropriate section before selecting a response.

Materials & experimental systems

n/a	Involved in the study	
☐	☒	Antibodies
☐	☒	Eukaryotic cell lines
☒	☐	Palaeontology and archaeology
☐	☒	Animals and other organisms
☐	☒	Human research participants
☒	☐	Clinical data
☒	☐	Dual use research of concern

Methods

n/a	Involved in the study	
☒	☐	ChIP-seq
☐	☒	Flow cytometry
☒	☐	MRI-based neuroimaging

Antibodies

Antibodies used	gammaH2AX, 1:1000, Millipore, 05-636 and 53BP1, 1:2000, Novus, NB100-304; p16INK4A, 1:50, Santa Cruz, sc-56330; p21Cip1, 1:200, Santa Cruz, sc-6246; p53, 1:200, Santa Cruz, sc-126; PCNA, 1:500, Abcam, ab18197, 1:200 Alexa-Flour 488, Invitrogen, 10424752 and 1:200 Alexa-Flour-568, Invitrogen, 10348072;
Validation	Validated by the suppliers

Eukaryotic cell lines

Policy information about cell lines

Cell line source(s)	All human-derived primary skin fibroblast cells were purchased from Coriell Institute (USA). Control fibroblasts included AG08498 (male, 1 year), GM22159 (male, 1 day), GM22222 (male 1 day), GM03349 (male, 10 years) and GM05757 (male, 7 years).
Authentication	Cell lines were not authenticated.
Mycoplasma contamination	All cell lines were tested free of mycoplasm.
Commonly misidentified lines (See ICLAC register)	No commonly missclassified cell lines were used.

Animals and other organisms

Policy information about studies involving animals; ARRIVE guidelines recommended for reporting animal research

Laboratory animals	18 male and 18 female C57Bl6/j mice aged between 38 and 88 weeks of age were used.
Wild animals	the study did not involve wild animals
Field-collected samples	study did not involve samples collected from the field
Ethics oversight	All animal experiments were approved by the Institutional Animal Care and Use Committee at MedImmune (Gaithersburg, MD, United States) and/or the Danish Animal Experiments Inspectorate (licenses 2017-15-0201-01378 and 2017-15-0201-01321) and performed in accordance with internationally accepted principles for the use of laboratory animals including the European directive 2010/63/EU on the protection of animals used for scientific purposes.

Note that full information on the approval of the study protocol must also be provided in the manuscript.

Human research participants

Policy information about studies involving human research participants

Population characteristics	The patient samples were retrieved from a pathological specimen repository. The selected specimen were gathered from individuals with a flat distribution of age with equal participation between age 20 and 80.
Recruitment	The individuals were sampled from patients for whom samples of naevi on non-sun exposed skin had undergone pathology without malignant findings at a major pathology department in Copenhagen. We selected patient samples from the Danish National Register of Pathology requisitioned in 2007-2017 and coded with one or more PatoSNOMED topology code: T02530 (Skin on penis), T76330 (Foreskin), T80200 (Mons pubis), T02471(Skin on nates), T02480 (Skin on abdomen), T02430 (Skin on breasts) and one or more procedure code: P30620 (resect), P306X0 (ectomy preparation), P30611 (excision biopsy) and one or more morphology code: M87400 (junction naevus), M87500 (dermal naevus), M87600 (compound naevus).
Ethics oversight	Work on human samples and clinical records were approved by the National Committee on Health Research Ethics (#H-19078472) and by the Danish Data Protection Agency (#514-0226/18-3000).

Note that full information on the approval of the study protocol must also be provided in the manuscript.

Flow Cytometry

Plots

Confirm that:

☒ The axis labels state the marker and fluorochrome used (e.g. CD4-FITC).

☒ The axis scales are clearly visible. Include numbers along axes only for bottom left plot of group (a 'group' is an analysis of identical markers).

☒ All plots are contour plots with outliers or pseudocolor plots.

☒ A numerical value for number of cells or percentage (with statistics) is provided.

Methodology

Sample preparation	Fibroblast cell lines AG08498, GM22159 and GM22222 were seeded in 6-well cell culture plates and 24 hours later DNA-damage induced senescence was conducted as described above (5 Gy, 10 Gy or 20 Gy). After 9 days cells were harvested by trypsinization and washed twice with PBS. Cells were fixed by adding dropwise ice-cold 70% ethanol while mixing the cells gently on a vortex mixer. Thereafter, the cells were incubated for 30 min on ice and washed twice in PBS. Fixed cells were incubated with RNase (100 µg/ml; ThermoFisher Scientific) at 37°C for 30 min. Propidium iodide (20 µg/ml; Sigma-Aldrich) was added and incubated for 30 min at 37°C.
Instrument	CytoFlex, Beckman Coulter
Software	Cytobank was used for analyses.
Cell population abundance	Roughly 30-70% of trypzinised were live according to gating and these were used for measuring cell cycle using propidium iodide. Data is shown as percent cells in each of the cycling phases which is how cell cycle data is always shown.
Gating strategy	Live cells were gated using forward- and side-scatter.

☒ Tick this box to confirm that a figure exemplifying the gating strategy is provided in the Supplementary Information.
